# A Bibliometric analysis of scientific publication on Peri-Implantitis from 1990 to 2020

**DOI:** 10.4317/jced.61551

**Published:** 2024-05-01

**Authors:** Saqib Ali, Beenish-Fatima Alam, Ali-Ahmad Alaithan, Mohammed-Ali Alnemer, Morooj Aljishi, Badr Al-Jandan

**Affiliations:** 1 Lecturer; Department of Biomedical Dental Sciences, College of Dentistry, Imam Abdulrahman Bin Faisal University, P.O Box, 1982, Dammam 31441, Saudi Arabia; 2 Associate Professor; Department of Oral Biology, Bahria University Medical and Dental College, Karachi, Pakistan; 3 Dental Student; College of Dentistry, Imam Abdulrahman Bin Faisal University, P.O Box, 1982, Dammam 31441, Saudi Arabia; 4 Dental Student; College of Dentistry, Imam Abdulrahman Bin Faisal University, P.O Box, 1982, Dammam 31441, Saudi Arabia; 5 Demonstrator; Department of Biomedical Dental Sciences, College of Dentistry, Imam Abdulrahman Bin Faisal University, P.O Box, 1982, Dammam 31441, Saudi Arabia; 6 Professor; Department of Biomedical Dental Sciences, College of Dentistry, Imam Abdulrahman Bin Faisal University, P.O Box, 1982, Dammam 31441, Saudi Arabia

## Abstract

**Background:**

Peri-implantitis can involve about 13% of implants and 20% of patients, it has been reported that its incidence increases from about 0.4 to 43.9% in 3–5 years. The purpose was to analyze, using bibliometric indicators, the scientific efficiency of different organization, countries, and researchers that published articles on Peri-implantitis in various dental journals during the period from 1990 to 2020.

**Material and Methods:**

The search was carried out using Scopus database on publications related to Peri-implantitis from 1990 to 2020 using VOSviewer 1.6.15. The selected search encompassed title of article, citation count, year of publication, authors, institution, country and keywords. Data maps were obtained from VOS viewer based on number of papers, citation count, sources, countries and authors. A density visualization analysis was performed to interpret the data. Bibliometric analysis with reference to citation and documents, authors, journals and keywords was also evaluated.

**Results:**

An upsurge in number of cumulative papers published on Peri-implantitis from 1990 to 2020 was observed. The top three countries that published most research papers on Peri-implantitis included United States, Sweden and Switzerland respectively. The most productive organization in the field was Blekinge Institute of Technology, Karlskrona, Sweden. The maximum numbers of papers were published in “Clinical Oral Implants Research”, while the most published and cited author was Niklaus P. Lang, with 50 papers, 5391 citations with 107.82 average citations per paper.

**Conclusions:**

There is a tremendous increase in number of publications on peri-implantitis through collaboration of authors, nations and institutes. Among the leading countries from where evidence is originating includes, USA, Sweden and Switzerland. The leading institutes whose work received most citations included, Kristiansand University (Sweden), Blekinge Institute of Technology (Sweden), Trinity college (Dublin) and University Of Bern (Switzerland). A positive trend of highly collaborative work was observed among the institutes and authors on peri-implantitis.

** Key words:**Bibliometric analysis, Peri-Implantitis, Scopus, Dental implants.

## Introduction

During the period of 1960s the concept of osseointegration was introduced by Per –Ingvar Brånemark (1), its application in dentistry has widely been used with the help of dental implants since then. Implant placements are annually estimated to be around 5 million in the United States and 15-20 million worldwide (2,3) with a 5 year success rate of 98.1% (4). However, as the placement of Osseointegrated implants has increased, so are the reports related to high rate of inflammatory response in peri-implant tissues. Peri-imlpantitis has raised concerns for clinicians and researchers as inflammation in adjacent structures of implant can lead to bleeding, swelling, suppuration, loss of bone and implant failure (5-8).

Peri-implantitis can involve about 13% of implants and 20% of patients, however it has been reported that its incidence increases from about 0.4 to 43.9% in 3–5 years (9,10). However, the incidence of this disease tends to vary among subjects. Regardless of principally being bacterial origin, various other factors such as presence of systemic disease, periodontal disease and smoking and may also increase the potential risk of developing peri-implantitis (11). Peri-implant inflammation can manifests as peri-implant mucositis, which establishes within the supporting soft tissues around an implant and is reversible (12). While Peri-implantitis is inflammatory loss of soft and hard tissue (bone) leading to irreversible damage around peri-implant tissues (13). Studies performed on the etiology of peri-implantitis reflects, that anaerobic bacteria in the plaque cause infection and inflammation of the surrounding soft and hard tissues resulting in bleeding and swelling of soft tissue, attachment loss, pocket formation, bone loss and implant mobility (14). Microbial adhesion to the surface of implant is an intricate phenomenon, that was initially defined as the “race for the surface” by Gristina *et al*, who defined the process that when bacteria occupies the implant surface in adequate numbers, the implant will get infested and may require removal (15).

Health of periodontal tissues plays a key role in the success or failure of the implant thus patients who are suffering with chronic periodontitis have higher incidence of developing peri-implantitis as compared to individuals with a healthy periodontal environment (16). Karoussis *et al*., conducted a research where patients were recruited for clinical and radiological assessment 10 years after the placement of dental implant. The patients had been treated for periodontal disease. The results of the study revealed that implants placed after chronic periodontal condition leading to loss of teeth showed lower survival rates as compared to implants placed after teeth loss due to trauma and decay (16).

Bibliometric analysis deals with various means used to retrieve and analyze quantifiable amount of data that has been published in research articles (17). The word “bibliometric” has been introduced in 1969 by Alan Pritchard that describes the usage of mathematical and statistical means to scrutinize journal, books and other sources. Various bibliometric analyses have been carried out on the different aspects of medicine and dentistry (18-21). In healthcare research, the significance of a disease condition and its scientific relevance is determined by the magnitude of research published in the form of peer-reviewed articles on the subject (22). These studies report aspects of a disease condition including, pathogenesis, diagnostic methods, progression, treatment methods, outcomes and prognosis, indicating knowledge and trends to improve efficiency of healthcare system (23). Research articles on Peri-implantitis have shown sequential increase in numbers from 1990-2020. This increase can be related to the development of new implant procedures and implant related complications that have increased over the past two decades. Furthermore there is a lack of bibliometric analysis specific to Peri-implantitis therefore, this study aims at identifying the changing trends, leading research centers, leading authors, countries and other related aspects in research related to Peri-implantitis.

## Material and Methods

-Search Strategy

Electronic search with the help of Scopus (https://www.scopus.com, Elsevier, Amsterdam, Netherlands) database was performed on January 1, 2021. The search topic was “Peri-implantitis” and search did not include any restrictions except the inclusion of original articles published from 1990 – 2020. The data obtained from Elseviers’s Scopus database included 2986 results, which after applying the exclusion criteria, the number of papers were further narrowed down providing 960 total papers. Two investigators reviewed the potential papers on Scopus database by screening the titles and abstract of the papers.

The selected search from Scopus encompassed title of article, citation count, year of publication, authors, institution, country and keywords. Data was exported from Scopus in csv format.

-Inclusion and Exclusion Criteria

Inclusion criteria for the study comprised 1) dentistry related papers, 2) Papers published in Peri-implantitis, 3) Papers written in English Language 4) Original Papers.

Exclusion criteria comprised of 1) subject specialty other than dentistry 2) reviews, book chapters, 3) Not Peri-implantitis related, 4) Animal studies.

-Data Analysis

The Visualization of Similarities viewer software (VOS viewer version 1.6.15 Centre for Science and Technology Studies) and Microsoft Excel spread-sheet was used to analyze the exported data from csv file. Set of data maps in the form of bubbles were obtained from VOS viewer which was based on number of papers, citation count, sources, countries and authors. Keywords clusters were analyzed with the help of density visualization. Visual analysis for number of publication was generated in the form of bubble and its association with other items was indicated by the distance between the bubbles. The bubbles were color coded for each item and had distinct meaning for each visualization.

Keywords effect with respect to its co-occurrence was identified by restricting a minimum number of 10 occurrences in the network visualization. Terms related to Peri-Implantitis, such as dental implants, tooth implant, peri-implantitis, adult, periodontitis, osseointegration were included. From the 5800 keywords 1021 met the threshold. A density visualization analysis was performed to reflect the data.

The percentage of the quantity was calculated by dividing document numbers from the total number of documents. Similarly, the average citation score per document was evaluated by dividing the citations by the total number of papers.

## Results

-Analysis of the Overall Growth Trend

**Figure 1:**
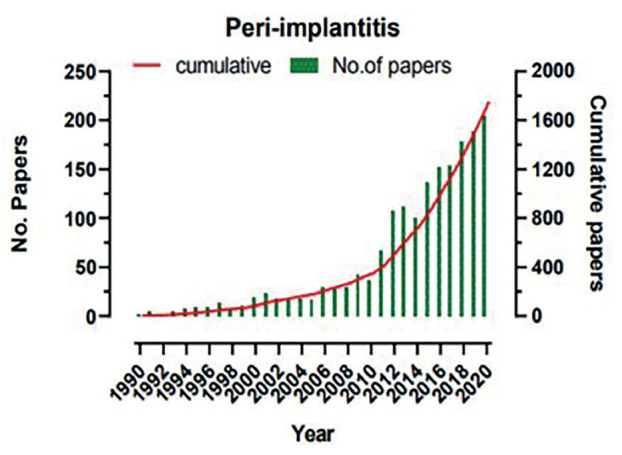
The total number of publications and cumulative publications on a yearly basis, yearly distribution and growth.

Figure 1 demonstrates the number of research papers published since 1990 to 2020 on Peri-implantitis. During early 90s the number of publication was quite low that is less than 10 papers, this trend continued till early 2000s. However, in 2012, a positive interest was developed and 125 papers were published. Similarly, in 2016 and 2018, about 175 and 190 articles got published while in year 2020, there were 200 published papers. Furthermore, the upsurge in number of cumulative papers suggests the greater interest of researchers in this field (Fig. 1) indicating that in 1990 and 1999 the cumulative papers that got published were 2 and 70 respectively. However, in the year 2000 and 2010 cumulative number of papers increased to 89 and 350 respectively and this increase in number of papers continued in the year 2018 with 1359 cumulative papers and in the year 2020 cumulative number of papers published were 1751, (Fig. 1).

-Leading Countries

 Table 1 highlights the top countries that published minimum of 25 papers related to Peri-implantitis. United States ranked first by publishing 202 papers which received 8726 citations. Although Sweden stood second by publishing 128 papers but these papers received 10271 citations, similarly Switzerland published only 99 papers but received 7965 citations. While Germany and Italy published 136 and 133 papers which received 5783 and 3476 citations respectively ( Table 1).

Interestingly Belgium received highest average citation for each paper published (32 papers) and received 93.25 average citations, while Switzerland and Ireland received 80.45 and 76.15 average citations respectively.

**Figure 2:**
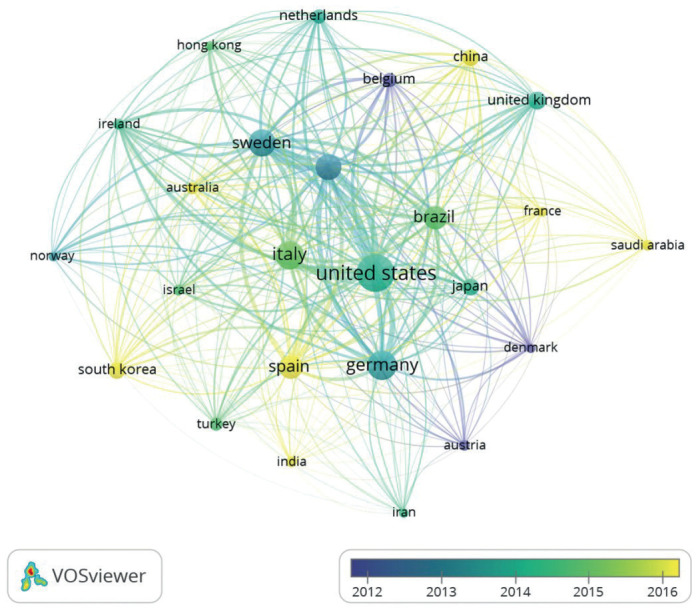
Country wise cooperative network on research related to peri-implantitis.

Total link strength provides an approximation of the collaborative research that has been carried out by a specific nation.USA, Sweden and Switzerland were among the highly collaborative countries having link strength of 2659, 2313and 2055, respectively. It was observed that researchers from USA has published papers with researchers in Switzerland, Sweden, Germany, Brazil, Italy, France, Belgium, and Ireland, (Fig. 2).

-Top Organisations

 Table 2 exhibits the prolific organizations that published highest research articles in relation to Peri-Implantitis. Kristiansand University from Sweden published 26 papers which received highest citations 3706 while the average citation of each paper was 142.54, followed by Blekinge Institute of Technology, Sweden that produced 24 papers and received 1133 citation, while average citation per paper was 47.21. Highest number of papers was published by the University of Bern, Switzerland (n=26) that received 890 citations. University of Michigan, USA published 20 papers receiving 252 citations with the average citation count of 12.6. Highest average citation per paper was received by Kristiansand University (121.69) followed by Trinity College Dublin (64.13). However Blekinge Institute of Technology had the highest total link strength (161) followed by Trinity College Dublin (142). University of Michigan had the lowest link strength, which were 36, ( Table 2).

-Authors and Co-author Relation

 Table 3 presents the authors that published the maximum papers on peri-implanttis. From 5386 authors, 15 authors met the threshold of publishing 20 articles. Authors Niklaus P. Lang published 50 papers that received 5391 citations and had the highest average citation per paper (107.82). While author Stefan Renvert produced 49 papers that received 3881 citations and had an average citation per paper of 79.20. However, Marc Quirynen published only 25 papers but had received the second highest average citation per paper of 93.60. Schwarz F. had the highest link strength among all the authors (865) followed by Renvert S. (778). The lowest number of papers was published (n=20) by Piattelli A, receiving 334 citations, with an average citation per paper of 16.7, ( Table 3).

-Leading Journals 

 Table 4 lists the major journals that published research documents associated with Peri-Implantitis. From the 140 journals, 19 journals published 15 articles related to this field. Results demonstrate that most preferred journal by authors was Journal of Clinical Oral Implants Research with 303 papers and 17545 citations. Journal of Clinical Periodontology published 128 articles and which received 6436 citations. International Journal of Oral and Maxillofacial Implants published 183 papers and acquired 1627 citations. While the Journal “Periodontology 2000” published 151 papers, acquiring only 1603 citations. Journal of Dental Research published 25 papers and received 1191 citations. Lastly, Brazilian Oral Research journal published 15 papers and acquired 78 citations, ( Table 4).

**Figure 3:**
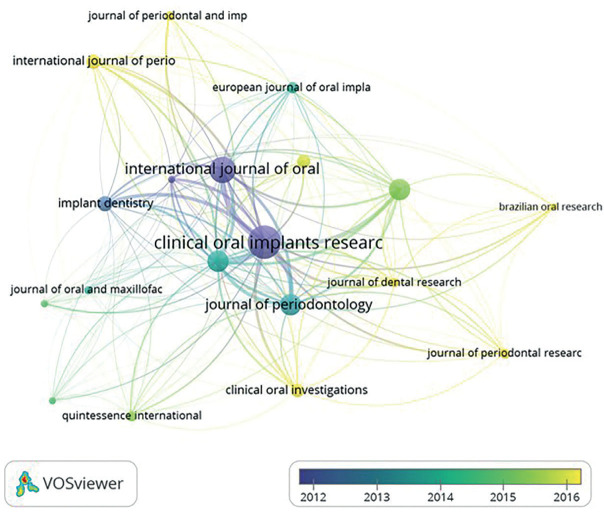
Journal and citation relationship of the sources that published research articles on Peri-implantitis.

Interestingly it can be noted that all the above mentioned journals had high JCR rank, with Periodontology 2000 having JCR rank of (7.7) followed by Journal of Clinical Periodontology and Journal of Periodontology having JCR rank of 5.242 and 3.742 respectively. Journals whose published papers received highest average citation was Periodontology 2000 being 106.9 followed by Clinical Oral Implants Research with 57.9 citations, ( Table 4, Fig. 3).

-Top Articles and their citation relationship

 Table 5 presents the top papers which had more than 200 citations. The top most cited article was published by Teughels W. (2006) receiving 660 citations followed by Jung R. E. (2008), which received 634 citations. Mombelli A. (1998) and Roos-Jansa˚ker A-M. (2006) received 430 and 416 citations respectively. Highest total link strength was achieved by papers published by author Roos-Jansa˚ker A-M. (2006a) which was 10, followed by Mombelli A. (2012) and Roos-Jansa˚ker A-M. (2006b) which ware 8 respectively.

Similarly paper by Teughels was also cited in papers published by different authors namely Mombelii A, Shibli Ja, ( Table 5).

- Keywords

**Figure 4:**
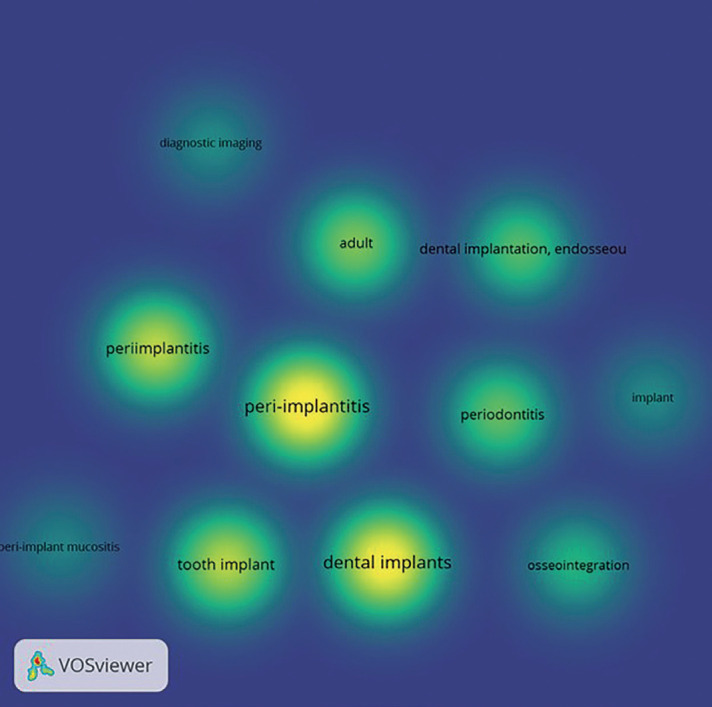
Density visualization of keywords used in relation to Peri-implantitis.

Figure 4 identifies the most frequently used keywords related to articles published on Peri-implantitis. The keywords were manually selected for generation of the density visualization map. The occurrence of the commonly used keywords were human (801) Peri-implantitis (699), dental implants (637), tooth implant (372), peri-implantitis (429), adult (341), periodontitis (247) and Osseo integration (206). The density visualization also identified other terms that have been related to this field such as periodontal pocket, dental prosthetic design, tooth implantations, surface property and microbiology, (Fig. 4).

## Discussion

This bibliometric analysis is based on identifying various articles published in the field of Peri-Implantitis. Peri-Implantitis primarily can be described as the inflammation of the soft and hard tissues associated with the dental implants (24). This condition if unresolved can lead to extensive destruction of the hard and soft tissues associated with the implant, however it further supplements the process of bone resorption, reduced osseo-integration and increase in the process of pocket formation (25,26).

For the purpose of bibliometric analysis Scopus database was utilized as it is a consistent and recognized database that provides a wide platform for peer reviewed literature. It helps and expedites the access to scientific data for researchers and at the same time support in utilizing the data for bibliometric analysis (27-29). Google scholar, another commonly used database tends to provide higher citation scores as compared to Scopus was not utilized as it uses different resources for the citation counts such as thesis, books, and dissertations (30). From the results of literature search conducted using the citations, from January 1990 to December 2020, a growing trend of publication on peri-implantitis could be visualized. This was further illustrated by the increase in the number of the cumulative papers over the period of time. This increase in number of paper can be attributed to multiple facts such as presence of high number of journals, number of author and research institute. However, it can be due to growing need of interdisciplinary research among various authors.

The highest number of published research as observed in this study originated from USA, these results are similar to previously conducted bibliometric analysis (31). USA has one of the highest allocated funds for research and development in the field of healthcare (32). In addition, these findings suggest that critical evidence related to the pathophysiology, diagnosis, etiology and management of peri-implantitis is originating from the USA. In addition, a high number of research evidence (n=198) on peri-implantitis is originating from Sweden. This is supported by the fact that there is an extensive custom of implant osseointegration research in Sweden, mainly due to the association of Dr. Branemark, the father of contemporary implantology and the creator of osseointegrated implants back in 1977 (33). Additionally many of the developing countries such as India and Iran have taken a keen interest in Peri-Implantitis and have started publishing papers that can be compared with the developed countries, demonstrating a positive trend on publications related to Peri-implantitis.

The present study revealed, Blekinge Institute of Technology in Karlskrona, Sweden, as the leading institution with highest number of research publications on peri-implantitis globally. These results are in contrast with the previously conducted bibliometric analysis, where University of Gothenburg in Sweden was most the productive organization (34). This could be credited to its robust research environment and the presence of prominent researchers and academics, such as Jemt, Berglundh, Albrektsson and Lekholm (35).

The most cited papers on peri-implantitis were authored by Teughels W and Jung R. E., where they made a significant impact in the field of implant dentistry and its complexities. Teughels *et al*. discussed the effect of material characteristics and surface topography on biofilm development. Biofilm which is composed of microorganism, its accumulation can be prevented by shedding lining epithelia multiple times a day in natural oropharyngeal tissues. However this may not be the case in prosthesis which is given to the patients in which natural removal of biofilm does not takes place and this may result in microorganism accumulation on the surface of hard tissues, implants, dentures leading to gingivitis, periodontitis and peri-implantitis (36).

Similarly a systematic review by Jung *et al*., was conducted on clinical performance of implant restorations and related biological and technical complications. In this article the author discussed about the single crown supported implant failure occurrence that were evident with the loss of more than 2mm bone adjacent to the implant surface indicating the presence of peri-implantitis (37). While Mombelli published a review article regarding the treatment options and diagnosis of Peri-implantitis. This study aimed at evaluating different parameters employed in diagnosis of Peri implantitis as well as making the right choices for its treatment (38).

NP Lang published numerous articles in collaboration with different authors namely Tord Berglundh, Marc Quirynen. Various papers published on Peri-implanitits in the journal “Clinical oral implants research” were highly cited by authors publishing articles in other journals such as Journal of Clinical Periodontology, Clinical Implant Dentistry and related research and the Journal of Periodontology (39-41). Similarly the highly cited article by Wim Teuguels was cited by many authors such as Marc Quirynen and Shibli Ja (36). Moreover these findings also highlight the fact that authors, journals and papers that receive high citations, is only possible by acknowledging the work of others and collaborating with different authors. This strong research network demonstrated with overlapping authorships show a trend of collaborative studies and research work among various research contributors.

The journal “Clinical Oral Implants Research” having impact factor 3.723, followed by the “Journal of Clinical Periodontology” having impact factor 5.241 published highest number of papers related to this topic. Similar results were achieved by previously conducted bibliometric analysis (37). This can be due to the fact that journals having highest impact factors tend to publish the highest number of research articles. In addition, keywords reveal the main focus of attention of a particular topic and provides path for incessant development of a particular topic. When performing literature search, keywords are useful in extracting appropriate data (42,43). The search for the most frequently utilized keywords was conducted to recognize the main topics within this field. The top keywords that were frequently used were dental implants, Peri-Implantitis, adult and Osseointegration.

The findings of the present study should be interpreted in light of the study limitations. In the present study rectification for self-citations was not performed, a lack of which by a journal or an author may aggregate more self-reference count and can lead to inaccuracy during the analysis (44). Moreover, citations that appear within different books and journals or those written in languages other than English were not included in this analysis. As the study presents a bibliometric analysis of published papers on peri-implantitis without an insight into the different aspects of etiology, diagnosis and management of the disease, further in depth analysis on the different aspects of peri-implantitis should be performed to ascertain disease development and management trends.

## Conclusions

The analysis suggests that there is a tremendous increase in number of publications on peri-implantitis through collaboration of authors, nations and institutes. Among the leading countries from where evidence is originating includes, USA, Sweden and Switzerland. The leading institutes whose work received the most citations included, Kristiansand University (Sweden), Blekinge Institute of Technology (Sweden), Trinity College (Dubin) and University Of Bern (Switzerland). A positive trend of highly collaborative work was observed among the institutes and authors on peri-implantitis.

## Figures and Tables

**Table 1:** Top countries publishing papers related to Peri-implantitis.

Country	No. of Papers	Citations	Average citations	Total link strength
Sweden	128	9422	73.61	2313
United States	202	8726	43.20	2659
Switzerland	99	7965	80.45	2055
Germany	136	5783	42.52	1506
Italy	133	3476	26.14	1175
Belgium	32	2984	93.25	582
Spain	81	2871	35.44	970
Brazil	76	2667	35.09	958
Ireland	26	1980	76.15	894
United Kingdom	42	1361	32.40	405
Netherlands	29	1229	42.38	373
Japan	39	873	22.38	372
Turkey	31	808	26.06	255
China	25	461	18.44	242
South Korea	30	373	12.43	235

**Table 2:** Top organization that published more than 15 documents.

Organization	Country	No. of papers	Citations	Average citations	Total link strength
Kristianstad University	Sweden	26	3706	142.54	207
Blekinge Institute of Technology	Sweden	19	1343	70.68	142
Trinity College	Ireland	12	1023	85.25	122
University of Bern	Switzerland	13	925	71.15	73
University of Michigan	USA	11	440	40.00	33
Universitätsklinikum Düsseldorf	Germany	10	286	28.60	21

**Table 3:** Top authors with that have published more than 20 documents.

Author	No. ofPapers	Citations	Average citations	Total Link Strength
Renvert S.	40	4167	104.18	423
Lang N.P.	28	3579	127.82	236
Salvi G.E.	21	2548	121.33	235
Schwarz F.	35	2245	64.14	350
Quirynen M.	16	2140	133.75	79
Berglundh T.	17	1625	95.59	122
Persson G.R.	16	1425	89.06	241
Becker J.	18	1192	66.22	260
Wang H.-L.	26	1149	44.19	164
Canullo L.	16	394	24.63	78

**Table 4:** Top journals.

Journals	Article published	Citations	Average citations	Total Link Strength
Clinical Oral Implants Research	170	14028	82.52	1355
Journal Of Clinical Periodontology	83	6115	73.67	946
International Journal Of Oral And Maxillofacial Implants	95	3478	36.61	482
Journal Of Periodontology	52	3152	60.62	491
Clinical Implant Dentistry And Related Research	73	2941	40.29	379
Implant Dentistry	26	611	23.50	185
International Journal Of Periodontics And Restorative Dentistry	40	542	13.55	180
Journal Of Periodontal Research	19	522	27.47	130
European Journal Of Oral Implantology	16	406	25.38	98
Clinical Oral Investigations	16	300	18.75	123
Journal of Oral Implantology	25	252	10.08	55
Quintessence International	18	244	13.56	62

**Table 5:** Top Papers with more than 200 citations.

Articles	Citations	Total Link Strength
Teughels W. (2006)	780	2
Jung R.E. (2008)	701	1
Roos-Jansåker A.-M. (2006b)	453	0
Karoussis I.K. (2003)	436	8
Mombelli A. (2012)	391	6
Leonhardt Å. (1999)	364	0
Buser D. (2012)	362	2
Brägger U. (2001)	318	0
Roos-Jansåker A.-M. (2006a)	302	3
Koldsland O.C. (2010)	298	5
Simonis P. (2010)	293	4
Derks J. (2016)	287	5
Ferreira S.D. (2006)	286	4
Ekelund J.-A. (2003)	285	1
Jepsen S. (2015)	282	2
Rosen P. (2013)	262	4
Costa F.O. (2012)	259	7
Serino G. (2009)	257	2
Hultin M. (2002)	242	4
Schwarz F. (2018)	241	6
Shibli J.A. (2008)	240	2
Quirynen M. (2006)	237	4
Leonhardt Å. (2003)	222	1
Dominguez Campelo l. (2002)	219	0
Karoussis I.K. (2004)	215	2

## Data Availability

The datasets used and/or analyzed during the current study are available from the corresponding author.
